# Supplementary material for: Tissue-resident Eomes+ NK cells are the major innate lymphoid cell population in human infant intestine
Source: Nat Commun. 2019 Feb 28;10:975. doi: 10.1038/s41467-018-08267-7 (PMC6395753; doi:10.1038/s41467-018-08267-7)
Supplement: Supplementary file 1 — Supplementary Information [file 41467_2018_8267_MOESM1_ESM.pdf]

Tissue-resident Eomes$^+$ NK cells are the major innate lymphoid cell population in human infant intestine

Sagebiel et al.

Supplementary Figures

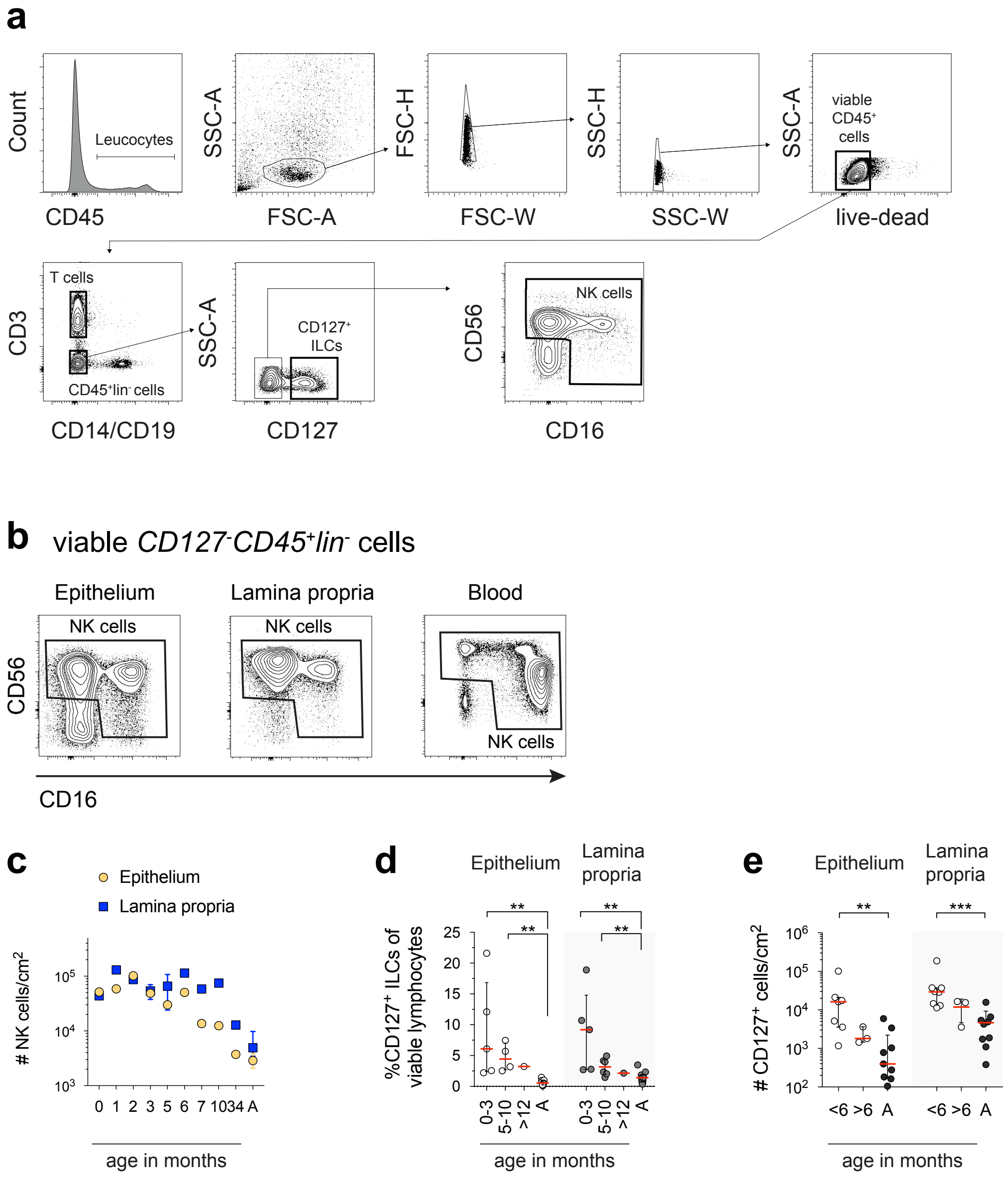

Supplementary figure 1 | ILC populations in intestines and blood

(**a**) Gating strategies for viable CD45$^+$ cells (Fig. 1a, 2a), CD45$^+$lin$^-$ cells (Fig. 1b, 2b, 7c), T cells (Fig. 8), CD127$^+$ ILCs (Fig. 7b, Supplementary Fig. 1d-e, 6) and NK cells (Fig. 2c-e, 3, 4, 5, 6, 7a, 7b, 8, Supplementary Fig. 1b-c, 2, 3, 4, 5). Fluorochromes may differ based on specific panel. (**b**) Representative flow diagrams of CD56 and CD16 expression by viable CD127$^-$CD45$^+$lin$^-$ cells in intestinal epithelium (EP), lamina propria (LP) and peripheral blood (B) of infants. (**c**) Absolute NK cell numbers/cm^2 at different ages in EP (yellow circles) and LP (blue squares) in infants and adults (infant samples N=9 (EP) and N=11 (LP), adult samples N=8 (EP) and N=9 (LP)). (**d**) Frequencies of CD127$^+$ ILCs of viable CD45$^+$ lymphocytes for different age groups in EP and LP samples (infant samples N=10 (EP) and N=12 (LP), adult samples N=11 (EP and LP)). (**e**) Absolute CD127$^+$ ILC numbers/cm^2 for different age groups in EP and LP samples (infant samples N=10 (EP) and N=11 (LP), adult samples N=9 (EP and LP)). Median frequencies indicated by red lines. Error bars define interquartile ranges between 75th and 25th percentiles. Statistical comparisons are Mann-Whitney U comparisons. Asterisks represent the following *p*-values: $^{**}p < 0.01$, $^{***}p < 0.001$. A = Adult samples. FACS plots of representative donors are shown.

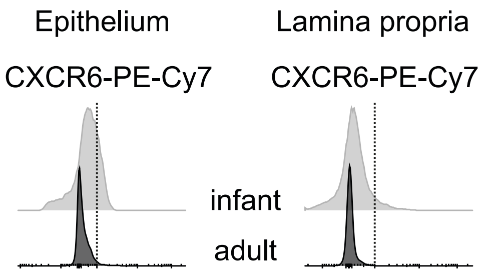

Supplementary figure 2 | CXCR6 expression by intestinal NK cells

Representative histogram overlay of CXCR6 expression by infant (gray) and adult (black) NK cells.

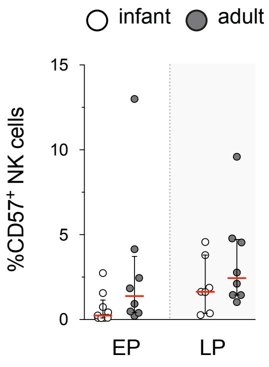

Supplementary figure 3 | Intestinal CD57+ NK cells are scarce

(**a**) Frequencies of CD57+ NK cells from epithelium (EP) and lamina propria (LP) of infants (white circles) and adults (dark circles) (infant samples N=9 (EP) and N=7 (LP), adult samples N=8 (EP and LP)). Median frequencies indicated by red lines. Error bars define interquartile ranges between 75th and 25th percentiles. Statistical comparisons are Mann-Whitney U comparisons.

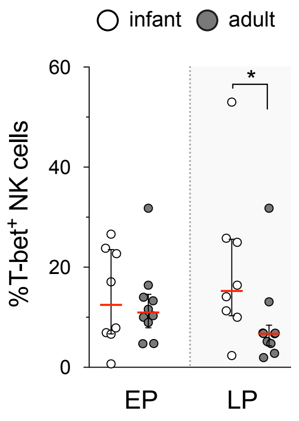

Supplementary figure 4 | T-bet expression in infant and adult intestinal NK cells

(**a**) Frequencies of T-bet$^+$ NK cells from epithelium (EP) and lamina propria (LP) of infants (white circles) and adults (dark circles) (infant samples N=8 (EP and LP), adult samples N=10 (EP and LP)). Median frequencies indicated by red lines. Error bars define interquartile ranges between 75th and 25th percentiles. Statistical comparisons are Mann-Whitney U comparisons. Asterisks represent the following *p*-values: $^*p < 0.05$.

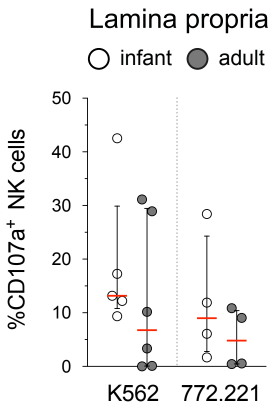

Supplementary figure 5 | Degranulation of intestinal NK cells upon stimulation

(**a**) Frequencies of infant (white circles) and adult (dark circles) CD107a$^+$ NK cells from lamina propria (LP). Cells were stimulated for 6 hours in the presence of K562 or 772.221 target cells (effector/target-ratio: 1:5) (infant samples N=5 (K562) and N=4 (772.221), adult samples N=6 (K562) and N=4 (772.221)). Median frequencies indicated by red lines. Error bars define interquartile ranges between 75th and 25th percentiles. Statistical comparisons are Mann-Whitney U comparisons.

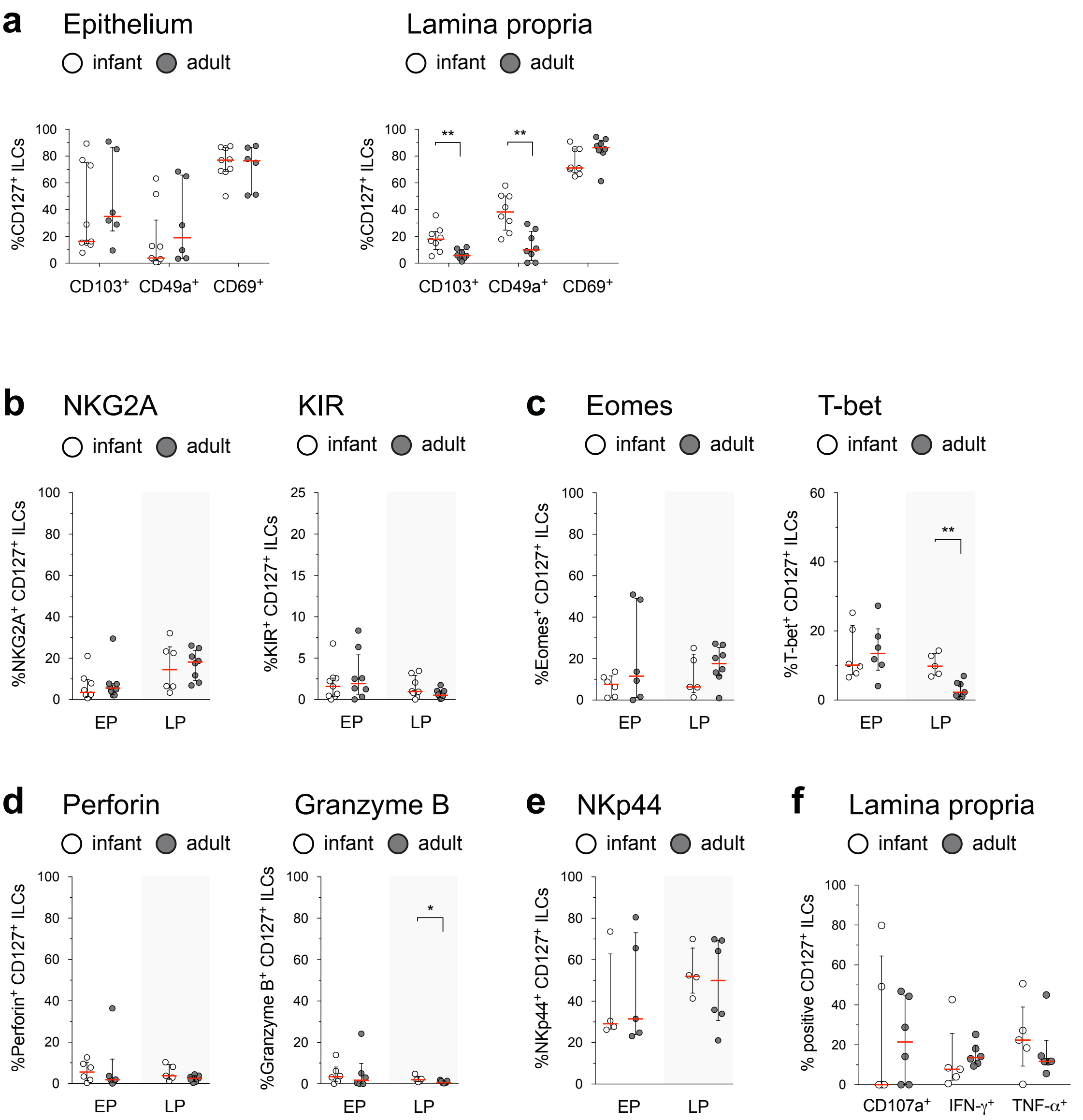

Supplementary figure 6 | Phenotypic characteristics of intestinal CD127$^+$ ILCs

(**a**) Frequencies of infant (white circles) and adult (dark circles) CD103$^+$, CD49a$^+$ or CD69$^+$ CD127$^+$ ILCs in epithelium (EP) and lamina propria (LP) tissues (infant samples N=9 (EP) and N=8 (LP), adult samples N=6 (EP) and N=8 (LP)). (**b**) Frequencies of infant (white circles) and adult (dark circles) NKG2A$^+$ or KIR$^+$ CD127$^+$ ILCs from EP and LP tissues (infant EP samples N=8 (NKG2A) and N=9 (KIR), infant LP samples N=6 (NKG2A) and N=8 (KIR), adult samples N=8 (EP and LP)). (**c**) Frequencies of infant (white circles) and adult (dark circles) Eomes$^+$ or T-bet$^+$ CD127$^+$ ILCs from EP (infant EP samples N=6 (Eomes and T-bet), infant LP samples N=5 (Eomes and T-bet), adult EP samples N=6 (Eomes and T-bet), adult LP samples N=8 (Eomes) and N=7 (T-bet)). (**d**) Frequencies of infant (white circles) and adult (dark circles) perforin$^+$ or granzyme B$^+$ CD127$^+$ ILCs from EP and LP tissues (infant samples N=6 (EP) and N=5 (LP), adult samples N=6 (EP) and N=7 (LP)). (**e**) Frequencies of infant (white circles) and adult (dark circles) NKp44$^+$ CD127$^+$ ILCs from EP and LP (infant samples N=4(EP and LP), adult samples N=5 (EP) and N=6 (LP)). (**f**) Frequencies of LP CD107a$^+$, IFN-ɣ$^+$ or TNF-α$^+$ CD127$^+$ ILCs in infants (white circles) and adults (dark circles). Cells were stimulated for 6 hours in the presence of PMA and ionomycin (infant samples N=5, adult samples N=6). Median frequencies indicated by red lines. Error bars define interquartile ranges between 75th and 25th percentiles. Statistical comparisons are Mann-Whitney U comparisons. Asterisks represent the following *p*-values: $*p < 0.05$, $**p < 0.01$.
