# Supplementary material for: Tissue-resident Eomes+ NK cells are the major innate lymphoid cell population in human infant intestine
Source: Nat Commun. 2019 Feb 28;10:975. doi: 10.1038/s41467-018-08267-7 (PMC6395753; doi:10.1038/s41467-018-08267-7)
Supplement: Supplementary file 3 — Reporting Summary [file 41467_2018_8267_MOESM3_ESM.pdf]

nature research

Corresponding author(s): Madeleine J. Bunders

Reporting Summary

Nature Research wishes to improve the reproducibility of the work that we publish. This form provides structure for consistency and transparency in reporting. For further information on Nature Research policies, see Authors & Referees and the Editorial Policy Checklist.

Statistical parameters

When statistical analyses are reported, confirm that the following items are present in the relevant location (e.g. figure legend, table legend, main text, or Methods section).

n/a	Confirmed	
☐	☒	The exact sample size (*n*) for each experimental group/condition, given as a discrete number and unit of measurement
☐	☒	An indication of whether measurements were taken from distinct samples or whether the same sample was measured repeatedly
☐	☒	The statistical test(s) used AND whether they are one- or two-sided<br>*Only common tests should be described solely by name; describe more complex techniques in the Methods section.*
☒	☐	A description of all covariates tested
☒	☐	A description of any assumptions or corrections, such as tests of normality and adjustment for multiple comparisons
☐	☒	A full description of the statistics including central tendency (e.g. means) or other basic estimates (e.g. regression coefficient) AND variation (e.g. standard deviation) or associated estimates of uncertainty (e.g. confidence intervals)
☒	☐	For null hypothesis testing, the test statistic (e.g. *F*, *t*, *r*) with confidence intervals, effect sizes, degrees of freedom and *P* value noted<br>*Give P values as exact values whenever suitable.*
☒	☐	For Bayesian analysis, information on the choice of priors and Markov chain Monte Carlo settings
☒	☐	For hierarchical and complex designs, identification of the appropriate level for tests and full reporting of outcomes
☒	☐	Estimates of effect sizes (e.g. Cohen's *d*, Pearson's *r*), indicating how they were calculated
☐	☒	Clearly defined error bars<br>*State explicitly what error bars represent (e.g. SD, SE, CI)*

Our web collection on statistics for biologists may be useful.

Software and code

Policy information about availability of computer code

Data collection	Flow cytometry data was collected using an BD LSR Fortessa with BD FACSDIVA Software v7.0 (BD Biosciences).
Data analysis	Flow cytometry data was analyzed using FlowJo v10 (TreeStar). Statistical analyses were done using GraphPad Prism 7. viSNE and SPADE plots were generated using Barnes-Hut t-distributed stochastic neighbor embedding (bht-SNE or viSNE) algorithm and SPADE clustering respectively, provided by the Cytobank platform (Cytobank Inc.)

For manuscripts utilizing custom algorithms or software that are central to the research but not yet described in published literature, software must be made available to editors/reviewers upon request. We strongly encourage code deposition in a community repository (e.g. GitHub). See the Nature Research guidelines for submitting code & software for further information.

Data

Policy information about availability of data

All manuscripts must include a data availability statement. This statement should provide the following information, where applicable:

- Accession codes, unique identifiers, or web links for publicly available datasets
- A list of figures that have associated raw data
- A description of any restrictions on data availability

Data used in this study have been collected in a clinical study and are subject to regulations of the Ethics Committee of the Ärztekammer Hamburg that approved

these studies. Participant's written consent has been provided to data generation and handling according to approved protocols. Data storage is performed by the HPI. A reporting summary for this Article is available as a Supplementary Information file. Data are available upon request from the corresponding author and can be shared after confirming that data will be used within the scope of the originally provided informed consent.

Field-specific reporting

Please select the best fit for your research. If you are not sure, read the appropriate sections before making your selection.

☒ Life sciences ☐ Behavioural & social sciences ☐ Ecological, evolutionary & environmental sciences

For a reference copy of the document with all sections, see nature.com/authors/policies/ReportingSummary-flat.pdf

Life sciences study design

All studies must disclose on these points even when the disclosure is negative.

Sample size	Sample sizes were determined based on previous studies on infant mucosal immune cells (Bunders et al., Blood 2012)
Data exclusions	Only non-inflammatory intestinal samples were included in this study
Replication	All data was conducted in multiple individual experiments. The number of individual experiments used for the generation of one figure is expressed in the figure legend.
Randomization	N/A
Blinding	Blinding was not performed for the acquisition and analysis of data.

Reporting for specific materials, systems and methods

Materials & experimental systems

n/a	Involved in the study	
☐	☒	Unique biological materials
☐	☒	Antibodies
☐	☒	Eukaryotic cell lines
☒	☐	Palaeontology
☒	☐	Animals and other organisms
☐	☒	Human research participants

Methods

n/a	Involved in the study	
☒	☐	ChIP-seq
☐	☒	Flow cytometry
☒	☐	MRI-based neuroimaging

Unique biological materials

Policy information about availability of materials

Obtaining unique materials	Human tissues were collected after donors (adults) or their guardians (infants) provided informed consent. Pediatric intestinal tissues were obtained during surgery to correct gastrointestinal congenital abnormalities and reconstruction of ileostomy. Adult samples were collected upon ileostomy reconstructions. All donors were free of inflammatory conditions. All tissues were obtained with approval of the ethics committee of the medical association of the Freie Hansestadt Hamburg (Ärztekammer Hamburg).

Antibodies

Antibodies used	The following monoclonal antibodies (all anti-human) were used for surface staining (clone, catalogue number, dilution): CD3-BUV395 (UCHT1, 563546, 1:80), CD56-BV786 (NCAM16.2, 564058, 1:100), CD16-BUV737 (3G8, 564434, 1:80), CD57-BV605 (NK-1, 563895, 1:160) from BD Bioscience. CD45-BV711 (HI30, 304049, 1:100), CD45-Alexa Fluor 700 (2D1, 368514, 1:80), CD14-PE-Cy7 (M5E2, 301814, 1:100), CD14-BV510 (M5E2, 301842, 1:100), CD19-PE-Cy7 (SJ25C1, 363011, 1:100), CD19-BV510 (HIB19, 302242, 1:100), CD127-PE-Dazzle594 (A019D5, 351336, 1:100), CD103-PE-Cy7 (Ber-ACT8, 350212, 1:100), CD69-BV711 (FN50, 310944, 1:80), CD69-BV605 (FN50, 310938, 1:50), CD69-BV421 (FN50, 310930, 1:200), NKp46/CD335-BV421 (9E2, 331913, 1:40), NKp44/CD336-PE (P44-8, 352107, 1:40), CXCR6/CD186-PE-Cy7 (K041E5, 356012, 1:20), CD107a-BV421 (H4A3, 328626, 1:40) from BioLegend. CD103-PerCP-eFluor710 (Ber-ACT8, 46-1037-42, 1:40), CD7-APC-eFluor780 (eBio124-1D1, 47-0079-41,

1:80) from eBioscience. CD94-FITC (REA113, 130-098-975, 1:40), NKG2A-APC (REA110, 130-098-809, 1:40), Nkp44-PE-Vio770 (2.29, 130-104-195, 1:40), KIR2D-PE (NKVFS1, 130-092-688, 1:160), KIR3DL1/DL2-PE (REA970, 130-095-205, 1:80), CD49a-APC-Vio770 (TS2/7, 130-101-406, 1:40) from Miltenyi Biotec. hNKp80-APC (239127, FAB1900A, 1:40) from R&D Systems.

The following monoclonal antibodies were used for intracellular staining (clone, catalogue number, dilution): T-bet-BV711 (4B10, 644819, 1:40), Perforin-PerCP-Cy5.5 (d9G, 308114, 1:40), IFN -FITC (B27, 506504, 1:40), TNF -BV605 (MAb11, 502936, 1:40), Granzyme B-FITC (GB11, 515403, 1:40) from BioLegend. Eomes-eFluor 660 (WD1928, 50-4877-42, 1:40) from eBioscience. Zombie Aqua™ Fixable Viability Kit (BioLegend, 423101) was used to determine cell viability.

Validation

All antibodies are commercially available and are validated for flow cytometry applications by the vendor.

Eukaryotic cell lines

Policy information about cell lines

Cell line source(s)

K562 cells were obtained from DSMZ. 772.221 cells were obtained from ATCC.

Authentication

K562 cell line was authenticated by DSMZ. 772.221 cells have been identified by the the appropriate Gene Technological Authority by fragment analysis.

Mycoplasma contamination

Cells were not tested for Mycoplasma contamination.

Commonly misidentified lines
(See ICLAC register)

Name any commonly misidentified cell lines used in the study and provide a rationale for their use.

Human research participants

Policy information about studies involving human research participants

Population characteristics

Human tissues were collected after donors (adults) or their guardians (infants) provided informed consent. Pediatric intestinal tissues were obtained during surgery to correct gastrointestinal congenital abnormalities and reconstruction of ileostomy. Adult samples were collected upon ileostomy reconstructions. All donors were free of inflammatory conditions.

Recruitment

Participants were recruited at the University Medical Center Hamburg-Eppendorf by Physicians of the Department of Pediatric Surgery as well as Department of General, Visceral and Thoracic Surgery. All donors provided informed consent and studies were approved by the ethical committee of the Ärztekammer Hamburg.

Flow Cytometry

Plots

Confirm that:

☒ The axis labels state the marker and fluorochrome used (e.g. CD4-FITC).

☐ The axis scales are clearly visible. Include numbers along axes only for bottom left plot of group (a 'group' is an analysis of identical markers).

☒ All plots are contour plots with outliers or pseudocolor plots.

☐ A numerical value for number of cells or percentage (with statistics) is provided.

Methodology

Sample preparation

Tissue and blood samples were transported at 4°C and processed in the laboratory within 6 hours after surgery. The mononuclear cell fraction was isolated from blood using a density gradient. Blood was diluted 1:1 with Hank's Balanced Salt Solution (HBSS; Sigma-Aldrich), then layered on top of BIOCOLL (Biochrom GmbH) and centrifuged. The mononuclear cell fraction was aspirated and washed with phosphate buffered saline (PBS). Intestinal tissues obtained at surgery were first washed with PBS to remove faeces and blood. The muscular layer was removed. The size of intestinal tissues were documented after removal of the muscular layer. Intestinal tissues were next cut into 0.5x0.5 cm segments and incubated for 2x 20 minutes, at 37° C with Iscove's Modified Dulbecco's Medium (IMDM; Thermo Fisher Scientific) supplemented with 5 mM ethylenediaminetetraacetic acid (EDTA; Sigma-Aldrich), 2 mM 1,4-dithiothreitol (DTT; Carl Roth GmbH + Co. KG) and 1% fetal bovine serum (FBS; Biochrom GmbH) to detach the epithelial layer. Supernatant was filtered through a 70 µm cell strainer to obtain a single cell solution. Epithelial lymphocytes were isolated by density gradient centrifugation using BIOCOLL (Biochrom GmbH). The remaining intestinal tissue was minced and digested for 2x 30 minutes at 37°C with IMDM (Thermo Fisher Scientific) supplemented with 1 mg/ml Collagenase D (Sigma-Aldrich), 1% FBS (Biochrom GmbH) and 1000 U/ml DNAse I (STEMCELL Technologies). Supernatant containing cells was filtered through a 70 µm strainer to obtain a single cell solution. Lamina propria lymphocytes were isolated from single cell suspensions using a Percoll gradient (VWR International); standard isotonic Percoll solution (SIP) was prepared by supplementing 100% Percoll with 10% 10X PBS, using an additional 1X PBS which resulted in 60% SIP solution. After isolation, the number of viable cells was counted using Trypan blue.

Instrument

BD LSR Fortessa v7.0 (BD Biosciences)

Software	For collection of data, the BD FACSDIVA Software (BD Biosciences) was used. For analyzation of data FlowJo v10 (Tree Star) software was used.
Cell population abundance	Cell sorting technique was not used in the study
Gating strategy	Viable CD56+CD127-CD45+lin- cell gating (called NK cells in the manuscript):<br><br>-CD45- events were excluded from the initial cell population. This was then gated by FSC-A vs SSC-A<br>-Doublets were excluded by FSC-H versus FSC-W and subsequent SSC-H versus SSC-W gate<br>-The remaining cell population was the gated on Zombie Aqua negative population for viable cells.<br>- Further CD3+, CD14+/CD19+ and CD127+ cells were excluded.<br>- NK cells were defined by gating on CD56+ and CD16+/- cells as shown in Supplementary Figure 1.<br><br>Viable CD127+CD45+lin- ILC gating (called CD127+ ILCs in the manuscript):<br><br>-CD45- events were excluded from the initial cell population. This was then gated by FSC-A vs SSC-A<br>-Doublets were excluded by FSC-H versus FSC-W and subsequent SSC-H versus SSC-W gate<br>-The remaining cell population was the gated on Zombie Aqua negative population.<br>-Further CD3+, CD14+/CD19+ were excluded<br>-CD127+ ILCs were then defined by gating on CD127+ cells as shown in Supplementary Figure 1.

☒ Tick this box to confirm that a figure exemplifying the gating strategy is provided in the Supplementary Information.

April 2018
